# Dual paraneoplastic syndromes in a patient with small cell lung cancer: a case report

**DOI:** 10.1186/1752-1947-5-318

**Published:** 2011-07-19

**Authors:** Kristin Coners, Scott E Woods, Michael Webb

**Affiliations:** 1 Bethesda Family Medicine Residency Program, 4411 Montgomery Road, Suite 200, Cincinnati, OH 45212, USA

## Abstract

**Introduction:**

We describe the case of a patient with small cell lung cancer and dual paraneoplastic syndromes involving adrenocorticotropic hormone and calcitonin. To the best of our knowledge, dual paraneoplastic syndromes involving these two hormones have not been previously reported in the literature.

**Case presentation:**

A 74-year-old Caucasian woman presented with a left hilar mass and metastatic disease in the liver and right adrenal gland. The patient complained only of intermittent diarrhea. Her laboratory values exhibited metabolic alkalosis with hypokalemia, hypocalcemia, hypomagnesemia, hypophosphatemia, and hyperglycemia.

**Conclusion:**

We discuss the work-up and treatment of the patient's unusual laboratory presentation with two concurrent paraneoplastic syndromes.

## Introduction

Although paraneoplastic syndromes occur commonly, dual paraneoplastic syndromes occurring simultaneously in the same patient are very rare. We describe the case of a patient with small cell lung cancer and dual paraneoplastic syndromes involving adrenocorticotropic hormone and calcitonin.

## Case report

A 74-year-old Caucasian woman presented to the Emergency Department (ED) at our hospital with acute onset of thoracic back pain. Her medical history included hypertension, hypothyroidism, a right hip replacement, and diffuse large cell lymphoma in 1985, which was treated successfully with chemotherapy and radiation. She was a previous smoker who had quit approximately five years earlier. Upon review of her systems, she complained only of some diarrhea that she had experienced intermittently for one year. Her medications included atenolol, hydrochlorothiazide, and levothyroxine. Her vital signs and physical examination in the ED were remarkable only for thoracic spine tenderness. X-rays revealed a compression fracture of her T9 vertebra (age indeterminate) and a new left hilar mass. A renal panel revealed the following abnormalities: potassium 2.7 mEq/l (lower limit of normal (LLN), 3.5 mEq/l), chloride 74 mEq/l (LLN, 101 mEq/l), bicarbonate 47 mM/l (upper limit of normal (ULN), 36 mM/l), glucose 198 mg/dl, blood urea nitrogen (BUN) 38 mg/dl (ULN, 20 mg/dl), and creatinine 1.3 md/dl (ULN, 1.2 md/dl), with normal levels of sodium (137 mEq/l), total calcium (9.1 mg/dl), and magnesium (1.7 mEq/L). A computed tomography scan showed a left hilar mass with metastatic disease in the liver and right adrenal gland. There was no evidence of a pathological fracture of her ninth thoracic vertebrae.

The patient was admitted to the hospital for hydration and electrolyte replacement. During her hospital stay, the patient's blood sugar was elevated, and she received insulin coverage. She also received an oral bisphosphonate. Additional diagnostic work-up included a stool culture and testing for *Clostridium difficile *toxins A and B, which were negative. Interventional radiology was performed, and a biopsy of one of the liver lesions was obtained. The patient was discharged to home five days after admission with instructions to discontinue hydrochlorothiazide and begin taking spironolactone 25 mg twice daily, alendronate 70 mg weekly, and metformin 500 mg twice daily. Her renal panel at the time of discharge showed significant improvement: sodium 142 mEq/l (normal), potassium 3.3 mEq/l (LLN, 3.5 mEq/l), chloride 102 mEq/l (normal), bicarbonate 32 mM/l (normal), glucose 132 mg/dl, BUN 35 mg/dl (ULN, 20 mg/dl), creatinine 1.0 md/dl (normal), and total calcium 8.1 mg/dl (LLN, 8.5 mg/dl). She was instructed to follow up with her oncologist for biopsy results.

The patient re-presented to the ED four days after her discharge with complaints of worsening diarrhea. Aside from an elevated blood pressure of 164/76 mmHg, her physical examination and vital signs were again unremarkable. Her renal panel now showed sodium 141 mEq/l, potassium 1.9 mEq/l, chloride 95 mEq/l, bicarbonate 30 mM/L, glucose 177 mg/dl, BUN 46 mg/dl, creatinine 1.4 md/dl, total calcium 6.2 mg/dl, ionized calcium 2.7 mg/dl (LLN, 4.6 mg/dl), magnesium 1 mEq/L (LLN, 1.4 mEq/L), phosphorous 2.1 mg/dl (LLN, 2.5 mg/dl). The pathology report diagnosed her tumor as small cell lung cancer (SCLC).

Our residency program admitted the patient for hydration, electrolyte replacement, and further work-up. Stool studies including ova and parasites, repeat culture, and *Clostridium difficile *toxin were negative. A gastroenterologist performed a flexible sigmoidoscopy and reported that the colonic mucosa appeared normal. Biopsies showed minimal and focal active inflammation consistent with focal active colitis of uncertain origin. The biopsy did not reveal any evidence of architectural distortion consistent with inflammatory bowel disease or any evidence of lymphocytic or collagenous colitis.

We conducted serum testing to uncover a possible paraneoplastic cause for the patient's symptoms. Her vasoactive intestine peptide, pancreatic polypeptide, gastrin, serum aldosterone, plasma renin activity, T4, thyroid-stimulating hormone, and 1,25-hydroxy vitamin D levels were all within normal limits. We did discover elevations in the patient's calcitonin (81.8 pg/ml; ULN, 4.6 pg/ml) and midnight cortisol (80.9 μg/dl; ULN, 10 μg/dl). Her adrenocorticotropin hormone (ACTH) level was 52 pg/ml (normal range, 6 to 58 pg/ml). We then performed an overnight 8 mg dexamethasone suppression test. The patient's cortisol failed to adequately suppress, with pre- and post-test values of 68.5 μg/dl and 65.5 μg/dl, respectively. Additional abnormalities included an intact parathyroid hormone level of 408 pg/ml (ULN, 65 pg/ml) and a 1,25-hydroxy vitamin D level of 14 ng/ml (LLN, 20 ng/ml).

We treated the patient with oral ketoconazole and somatostatin (initially every eight hours subcutaneously and later somatostatin long-acting release intramuscularly). The oncology department also began chemotherapy with etoposide and carboplatin. The patient's diarrhea did improve, as did her laboratory values. Her calcitonin decreased to 31.3 pg/ml, and her midnight cortisol fell to 19.4 μg/dl. Unfortunately, the patient's condition deteriorated with the initial chemotherapy, and she chose to discontinue therapy. She was discharged to hospice care.

## Discussion

SCLC is the most common cancer histology associated with paraneoplastic syndromes. Paraneoplastic syndromes are divided into two categories: ectopic production of biologically active proteins produced by the cancer cells and cell-mediated immune responses targeted against neural tissue (Table 1). Patients can present with multiple paraneoplastic syndromes at the same time, especially when the tumor arises from neuroendocrine cells. Although rare, there have been reports in the literature of patients with two or more paraneoplastic syndromes involving SCLC [1-5]. We also found a single report of a patient with two sequential paraneoplastic syndromes concurrently with SCLC [6]. Our case, however, is the only one reporting SCLC secreting both ACTH and calcitonin at the same time.

**Table 1:** Paraneoplastic syndromes associated with small cell lung cancer^a^

	Ectopic hormone-associated syndromes		Immune-mediated neurologic syndromes		
	
Clinical syndrome	Incidence	SCLC hormone	Incidence	Antibody	SCLC-expressed gene or protein
Ectopic Cushing's syndrome	5%	ACTH			
Hyponatremia of malignancy	15%	AVP, CRH (rare)			
Hypertension	<1%	Renin			
Amenorrhea, galactorrhea	<1%	Prolactin, GH			
Hyperamylasemia	<1%	Salivary anylase			
					
Lambert-Eaton myasthenic syndrome			1%	Anti-VGCC	Synaptotagmin, MysB
Encephalomyelitis			<1%	Anti-Hu	HuD, HuC, Hel-N1, N2
Sensory neuropathy			<1%	Anti-Hu	HuD, HuC, Hel-N1, N2
Cerebellar degeneration			<1%	Anti-Hu	HuD, HuC, Hel-N1, N2
				Anti-VJCC, MysB	Synaptotagmin
				Anti-Ri	Nova-1
				Anti-Yo	CDR-34
Retinopathy			<1%	Anti-CAR	Recoverin
Stiff-person syndrome (encephalitis)			<1%	Anti-amphiphysin	Amphiphysin
Opsoclonus, myoclonus			<1%	Anti-Hu	HuD, HuC, Hel-N1, N2
				Anti-Ri	Nova-1
					HuD, HuC, Hel-N1, N2
					Synaptotagmin
					Nova-1

^a^SCLC, small cell lung cancer; ACTH, adrenocorticotropin hormone; AVP, arginine vasopressin; CRH, corticotropin-releasing hormone; GH, growth hormone; VGCC, voltage-gated calcium channel; MysB,; HuD, Human Neuronal Protein D; HuC, Human Neuronal Protein C; Hel-N1,; VJCC,; Ri,; Yo,; CDR-34,; anti-CAR, anti-coxsackie adenovirus receptor.

Reprinted with permission from Gandhi L, Johnson BE: **Paraneoplastic syndromes associated with small cell lung cancer**. *J Natl Compr Canc Netw *2006, **4:**631-638 [12].

We believe that two paraneoplastic syndromes derived from SCLC affected this patient (ACTH and calcitonin). Ectopic ACTH secretion caused new-onset diabetes mellitus and likely contributed to hypokalemia, metabolic alkalosis, thoracic compression fracture, hypertension, and emotional liability. Because of a midnight cortisol level of 80.9 μg/dl and a high index of suspicion, we bypassed a 24-hour urinary cortisol test and performed an overnight 8 mg dexamethasone suppression test to determine the source of her hypercortisolism. The patient's cortisol level failed to suppress by 68%, and we made a presumptive diagnosis that her tumor was secreting ectopic ACTH. Given her high normal ACTH values, we did attempt to check her ACTH level by radioimmunoassay to evaluate for the presence of "big ACTH" particles not identified by immunoradiometric assay. Unfortunately, the laboratory mistakenly ran the wrong study. We also wished to obtain a corticotropin-releasing hormone level, but the patient elected hospice care prior to this hormone being drawn.

In addition to apparent ectopic ACTH, we further postulate that ectopic secretion of calcitonin contributed to the patient's profuse diarrhea. SCLC secreting calcitonin is extremely rare but has been reported [7]. Breast cancer and medullary thyroid cancer more commonly secrete calcitonin. The patient's diarrhea had started one year prior to her initial presentation and worsened substantially before her hospital admission. In total, the patient had four tests for *Clostridium difficile*, two stool cultures, and one test for ova and parasites, all of which were negative. She had a normal endoscopy, and her pathology report found focal active colitis of unclear etiology. There was no evidence to suggest inflammatory bowel disease, and the patient had not been taking medications that would have caused this pathology.

Calcitonin has been known to cause diarrhea in the spectrum of Verner-Morrison syndrome. We believe that our patient did not display all of the electrolyte abnormalities generally seen in patients with this syndrome (hypokalemia, achlorhydria, metabolic acidosis, and hypercalcemia) because of ectopic ACTH secretion and previous treatment with a bisphosphonate. More specifically, the patient's diarrhea should have produced metabolic acidosis; however, she possessed a metabolic alkalosis with a δ-gap of 46 caused by excessive cortisol secretion and dehydration. In addition, the patient's hypokalemia was secondary to severe alkalosis, diarrhea, and excessive cortisol secretion. The patient's hypocalcemia resulted from increased gastrointestinal losses, acute critical illness, vitamin D deficiency, and renal insufficiency with secondary hyperparathyroidism.

When surgical therapy is not an option, ketoconazole is the best therapy for treating patients with SCLC and ectopic ACTH secretion [8]. Ketoconazole therapy results in biochemical and hormonal improvement for most patients with excessive cortisol secretion [9]. It has few adverse effects, but may impair the cortisol response to stress. After our patient's treatment with ketoconazole and somatostatin, her diarrhea did improve, as did her laboratory values. We feel that treatment with ketoconazole and somatostatin did benefit the patient, given that chemotherapy had failed to substantially reduce her tumor burden as demonstrated on subsequent imaging.

SCLC accounts for approximately 15% of all bronchogenic carcinomas [10]. The average age at diagnosis is 71 years. About 30% of patients with SCLC have limited-stage disease (cancer limited to one hemithorax and lymph nodes on the same side of the chest). Patients with limited-stage disease have a median survival approaching two years and a 14% five-year survival rate. Patients with extensive disease have a median survival of less than one year [10]. Patients with SCLC and ectopic ACTH secretion tend to have more extensive disease and exhibit less response to chemotherapy, and they are more likely to die prematurely [11].

## Consent

Written informed consent was obtained from the patient for publication of this case report and any accompanying images. A copy of the written consent is available for review by the Editor-in-Chief of this journal.

## Competing interests

The authors declare that they have no competing interests.

## Authors' contributions

KC collected all of the patient data and wrote the initial draft of the manuscript. SW checked all of the data for accuracy and did considerable writing and formatting of the manuscript for publication. MW made sure that all appropriate laboratory studies were performed for a precise diagnosis and reviewed the manuscript for accuracy. All authors approved the final manuscript.
