# Correction to: Dual paraneoplastic syndromes in a patient with small cell lung cancer: a case report

**DOI:** 10.1186/s13256-023-04217-0

**Published:** 2023-10-31

**Authors:** Kristin Conners, Scott E. Woods, Michael Webb

**Affiliations:** Bethesda Family Medicine Residency Program, 4411 Montgomery Road, Suite 200, Cincinnati, OH 45212 USA

**Correction** **:** **Journal of  Medical Case Reports 2011, 5:318** **https://doi.org/10.1186/1752-1947-5-318**

Following publication of the original article [1], the authors identified an error in the author name of Kristin Conners.

The incorrect author name is: Kristin Coners

The correct author name is: Kristin Conners

The author group has been updated above and the original article [1] has been corrected.
